# Substance use amongst secondary school students in a rural setting in South Africa: Prevalence and possible contributing factors

**DOI:** 10.4102/phcfm.v8i2.934

**Published:** 2016-04-08

**Authors:** Takalani G. Tshitangano, Oni H. Tosin

**Affiliations:** 1 Department of Public Health, University of Venda, South Africa

## Abstract

**Introduction:**

This study determined the prevalence of substance abuse amongst rural secondary school learners in a selected province of South Africa.

**Methodology:**

The study adopted a quantitative approach using a descriptive survey design. Data were collected using a self-administered questionnaire from a total of 338 randomly selected learners, age 14 to 18 years, from 10 secondary schools that make up a rural Vhembedzi circuit in the Limpopo Province. Permission to enter the circuit and the schools was obtained from the circuit manager and parents’ or learners’ informed consent was obtained prior to data collection.

**Results:**

The majority of the participants (94% male, 98% female) had never used substances. Most of the learners started using substances between the ages 15 to 20 years. The majority of learners who were using substances were male. Of the respondents, all the female (100%) students reported to have stopped substance abuse. The majority (63% male, 50% female) of the learners tried to stop substance abuse but failed. Most of the learners (72% male, 71% female) did not have family members who were substance users. The majority of the students attested that substances can be easily obtained in their communities or villages. The majority (68%) of the leaners knew that substance abuse is dangerous to health.

**Conclusion and recommendation:**

Rural secondary school learners in South Africa have a low prevalence rate of substance abuse. Hence, there is a need for a counselling program in each school to provide support and refer such learners to an appropriate institution for rehabilitation.

## Introduction

Substance abuse by people in all parts of the world, particularly adolescents, has long been of scientific, political and public concern.^1,2^ This concern is due to the potential short- and long-term adverse effects associated with the use of substances such as cigarettes, drugs, cannabis (dagga) etc., on individual well-being.^1,3^ Various studies have revealed that substance use amongst adolescents may lead to poorer health and negative social consequences. For instance, substance abuse is associated with unintentional injuries, cancer, homicides and suicides, depression, personality disorder, unplanned sexual activity and increased sexually transmitted diseases. Moreover, substance abuse has also been documented to contribute to the high rate of school dropout, unemployment, high level of crime as well as poverty, which in turn affects the economy of a country.^4,5,6^ According to the United Nations, globally, cannabis (dagga) is a widely consumed illicit drug. Although it is not the primary drug of abuse in most nations such as Europe, America, Australia or Asia, it has been found to be the primary drug of abuse in Africa, especially amongst young people.^7^

However, worldwide different studies have presented evidence of substance abuse amongst high school learners. For instance, according to the World Drug Report, a survey carried out by the USA government in 2010 amongst grade 10 learners showed a prevalence rate of 1.3% of young people who had ever used heroin.^7^ A Monitoring the Future survey of drug use and attitudes amongst American 8th, 10th, and 12th graders in 2014 indicates positive news about youth drug use, including: decreasing use of alcohol, cigarettes, and prescription pain relievers; no increase in use of marijuana; decreasing use of inhalants and synthetic drugs, and a general decline over the last two decades in the use of illicit drugs. However, the survey underlined concerns over the high rate of e-cigarette use and easing attitudes around some types of drug use, particularly decreases in perceived harm and disapproval of marijuana use.^8^ In Canada, a study conducted in 2008 and 2009 amongst grade 10 to 12 learners indicated that 2.3% of these pupils had used heroin and 1.4% had used it once in the past month, whereas it is documented that, in South Africa, the use of heroin amongst age 13 to 22 years was 6.2%. With regard to cannabis (dagga) abuse, a study conducted in Kenya in 2007 revealed that 1.1% of adolescents of age 15 to 17 years had used cannabis. The World Health Organization (WHO) conducted a survey in Zambia amongst grade 7 to 10 learners. Findings showed that cannabis abuse was 35.5% amongst the learners. Meanwhile, from a survey conducted in South Africa in 2008 amongst young people of age 13 to 22 years, the rate of cannabis abuse was 12.7%, whilst in the USA cannabis usage by grade 10 pupils in 2010 was 33.4%.^7^

In South Africa, substance abuse is extremely serious, with drug usage reported as being at twice the world norm. Over 15% of the population suffers from a drug problem.^6,9^ The figures published by the South African Police Service show that drug abuse accounts for 60% of all crime in the country.^6^

In order to combat the above problem, the government of South Africa has put in place, and strengthened, policies on drug control. This policy includes: *The South African Drugs and Drug Trafficking Act* (140/1992) that was replaced by the *Prevention of and Treatment for Substance Abuse Act* 70 of 2008, promulgated on 01 April 2013. This Act covers new challenges regarding the prevention of drug abuse and addresses gaps which existed in the Act of 1992. Its aims are to provide a comprehensive national response for the combating of substance abuse; to provide mechanisms aimed at demand and harm reduction in relation to substance abuse through prevention, early intervention, treatment and re-integration programmes.^10^ In addition, the government has also been working assiduously with various sectors such as the health sector, educational sector and non-government organisations in the country to prevent substance abuse, particularly amongst adolescents. For instance, the Department of Education has implemented a ‘Revised Curriculum 2005 initiative’ that includes a Life Orientation Area of Learning consisting of components that address adolescent risk behaviours, such as drug use and teenage sexuality, as part of a holistic initiative with the intention of promoting healthy development of young people. The substance abuse section was reviewed and emphasised in order to address the issue of drug abuse problem within South Africa. In addition, the department also established a ‘Policy Framework for the Management of Drug Abuse by Learners in Schools and Further Education and Training Institutions’ so as to serve as a guide to all schools in developing substance abuse policy. The policy instructs both teachers and parents of the learners to be educated about substance abuse.^11^

Also, local studies have shown evidence of drug abuse amongst high school learners in various part of the country. In a study by Onya and Flisher, amongst rural high school students in Mankweng, Limpopo Province, South Africa, the researchers documented that the prevalence rates for previous month (recent) use of alcohol, cigarettes, cannabis, glue and spirits were 6.4%, 10.5%, 1.4%, 1.2% and 0.8% respectively.^12^ It was further stated that, for all substances, males had higher prevalence rates than females; this is also supported by a study carried out in Nigeria by Oshodi et al.^13^ Onya and Flisher attested that there was a lower prevalence rate of substance use amongst black Pedi high school students compared to other studies. Most of these studies were carried out to determine if the government as well as Department of Education efforts to prevent substance abuse amongst learners are effective, but there is no published study on this subject in Vhembe district. Thus, this study aimed to fill the gap.

## The purpose of the study

This study aimed to establish the prevalence and the age of debut as well as possible contributing factors to substance abuse amongst rural secondary school learners in Vhembe district and north of Kruger National Park, Limpopo Province, South Africa.

## The study objectives

To assess the prevalence level and the age of debut of substance abuse amongst secondary school learners.

To determine the factors possibly contributing to substance use amongst secondary school learners.

## Methodology

### Study design

Following the purpose of the study, a quantitative cross-sectional descriptive survey design was adopted. Since the study revolves around risk behaviour-related practices, a descriptive research design is deemed suitable by the researchers because it describes and interprets phenomena that are in existence, whilst at the same time using a cross-sectional survey method to collect data from subjects at one point in time to describe a phenomenon.^14^ Cross-sectional design also involves a once-off administration of the survey instrument to a sample. Therefore, it yields data on the desired variables as they existed at the time of the survey.

### The study setting

The study was carried out at the Vhumbedzi educational circuit that is located in the east of Sibasa in the Vhembe district and north of Kruger National Park. The circuit consists of 10 secondary schools, 24 primary schools and one independent primary school. The target population of this project is all school learners from grade R to grade 12 in all the 26 circuits of Vhembe district. The following are Vhembe district circuits: Central; Dzindi; Dzondo; East; Luvhuvu; Mudaswali; Mutshindudi; Mvudi; Ndzhelele east; Ndzhelele west; Niani; Hlanganani central; Soutpansberg north; North east; Sambandou; Sekgosese north; Sibasa; South; Tshilamba; Tshinane; Vhumbedzi; Vhurhonga; Vhurhonga and West. Vhumbedzi circuit and secondary school learners were phase one population of the study.

## Population and sample

### Target population

Ten secondary schools in the Vhumbedzi educational circuit with a total population of 5019 learners were involved in the study. The population comprised grades 8 to 12 male and female learners.

### Sample selection and procedure

Based on the sampling frame of 5019, sample size of *n* = 370 was calculated using Slovin’s formula (*n* = N/[1+Ne^2^]) where *n* and *N* denote the sample and population sizes respectively, thus allowing a margin error of *e* = 0.05.

A two-stage stratified sample selection process was employed using grades and gender as strata within each of the 10 participating schools. Learners were randomly selected within each stratum based on population proportional to size procedure that ensured proportional representativeness of grade and gender in the final sample.

### Instrument

The instrument was adapted from the 2011 high school Youth Risk Behaviour Survey (YRBS) of the Centers for Disease Control and Prevention.^15^ It was a self-administered questionnaire and semi-structured with closed and open-ended questions. The open-ended questions were included to capture a variety of responses so as to enhance and enrich the quantitative data. Whereas the instrument was written in English and required approximately 60 min to complete, caution was taken to ensure that it was user-friendly and understandable. The questionnaire was divided into three sections as follows:

- Demographic profile of the participants.
- Prevalence rate of substance abuse and age of debut amongst the learners.
- Possible contributing factors to substance abuse.

A Likert scale format and ‘yes’ or ‘no’ response options were incorporated in the instrument to collect responses to some of the questions in the instruments.

### Instrument validity

To ensure validity, the instrument was adapted from the YRBS questionnaire of the Centre for Disease Control and Prevention to suit the local conditions.^15^ A wide range of literature was also consulted on the variables of interests. Also, the instrument was pre-tested on some volunteer learners in a school similar to the target population. Pre-testing results were used to rephrase and modify some aspects of the questionnaire thus making it suitable and comprehensible to the participants.

### Instrument reliability

The reliability of the instrument was bolstered by adapting a questionnaire based largely upon the Centres for Disease Control and Prevention 2011 national high school *Youth Risk Behaviours Survey*. The YRBS is a standardised instrument developed by the Centre for Disease Control to measure risk behaviours of high school students with a generally high reliability rating (Kappa = 61% – 100%).^15^

### Data collection or survey procedure

The study was conducted over a 3-week period between October and November 2012. All 10 schools were visited by the research team to identify the learners who were to participate in the study. Dates for data collection were pre-arranged by the circuit office and school authorities. A special class was organised with each participating school where the research team briefed the participants and assisted in facilitating the administration of the instrument and addressing any emerging issues. The administration of the questionnaires lasted approximately 60 min.

### Data analysis

Survey responses were coded and analysed by using the Statistical Package for the Social Sciences (SPSS) version 21.0 software and Microsoft Excel. Descriptive statistics (percentages, mean etc.) were used to summarise the data.

## Ethical procedure

The Research and Innovation Directorate of the University of Venda issued an ethical clearance certificate (SHS/12/PH/03/ 0812) for the approval of the study in August 2012. Permission to conduct the research and to enter schools was obtained from the Department of Health, Limpopo Province, and the Vhumbedzi circuit office. Final access to the participating schools was negotiated with the school authorities. Written informed consent was obtained from the participants and their parents before the administration of the instrument. Anonymity, confidentiality and voluntary participation were assured. In addition, participants’ names and identities were not required; at the same time, no staff member was allowed at the survey venue during the time the questionnaires were administered.

### Limitation

The study involved 10 secondary schools in only one of the 26 educational circuits of the Vhembe district and as such generalisation of the result is not possible. An additional limitation could be biased by subjective responses that could arise from the sensitivity of a study aimed at soliciting evidence of violence-related behaviour of participants and their socioeconomic status.

## Results

The results are presented according to the objectives of the study as follows.

### Prevalence level and the age of ebut of substance abuse

The findings of this study revealed that the majority of the male learners (94%, *n* = 135) said they had never used substances, and a very few (6%, *n* = 8) attested to have used substances such as cigarettes, drugs, dagga etc. Similarly, the majority of the female learners (98%, *n* = 168) said ‘no’ when asked if they had ever used substances, whilst a very few (2%, *n* = 3) said they had used substances. Meanwhile, the majority (67%, *n* = 6) of the male learners started using substances between the age of 13 to 15 years, whereas the female students mostly began substance use between the age of 16 to 18 years (33%, *n* = 2). Table 1 shows the age at debut of substance use:

**TABLE 1:** Distribution of respondents according to age at debut of substance use.

Variable	Male		Female	
	
	Number	%	Number	%
**Age at Debut of Substance Use**				
5–10	0	0.00	1	25.00
10–15	3	33.33	0	0.00
15–20	6	66.67	1	25.00
20–25	0	0.00	2	50.00
**Total**	**9**	**100.00**	**4**	**100.00**

Surprisingly, the majority of the male learners (63%, *n* = 5) said they were still using substances, and a few (37%, *n* = 3) said they were no longer using them, whereas all the female learners said they had stopped using substances. When further asked if they have ever tried to stop substance use and failed, a few (37%, *n* = 3) of the male learners said ‘yes’ and most (63%, *n* = 5) said ‘no’. Meanwhile, only 50% (*n* = 1) of the female learners attested to have tried to stop substance use but failed and also only 50% (*n* = 1) said ‘no’ to this question.

### Possible factors contributing to substance use

Of the male learners, most (72%, *n* = 108) said they did not have substance users in their families and a few (14%, *n* = 21) attested to having a family member who used substances. Likewise, the majority (70%, *n* = 124) of the female students said they had no family members using substances and a few (18%, *n* = 32) indicated having family members who used substances. Some of the male learners (15%, *n* = 22) and 12% (*n* = 21) of the female students were unsure if any of their family members were substance users.

The majority of the male (54%, *n* = 82) and female (72%, *n* = 129) learners said they did not have friends or colleagues who used substances, and some of the male (25%, *n* = 35) and female learners (7%, *n* = 13) confirmed having friends or colleagues who were substance users. Whereas, a few of the male (23%, *n* = 34) and female (20%, *n* = 36) students were unsure if they had friends who were substance users.

Of the total respondents, some of the learners, both male (48%, *n* = 66) and female (47%, *n* = 85), said substances were not used in their schools, whilst some male (43%, *n* = 65) and female (29%, *n* = 53) learners said that substances were used in their schools. Only a few of the male (13%, *n* = 20) and female (23% *n* = 42) learners were unsure of substance usage in their schools.

The majority of the learners, male (48%, *n* = 72) and female (39%, *n* = 69), attested that substances were easy to obtain in their community villages. Of the learners, 30% (*n* = 45) males and 31% (*n* = 55) females said that it was not easy to obtain substances in their communities. Whereas, 21% (*n* = 32) male and 31% (*n* = 55) female learner were unsure.

Most of the male (68%, *n* = 100) and female (66%, *n* = 115) learners were aware that substance abuse is dangerous to health. Some male (23%, *n* = 34) and female (26%, *n* = 45) learners did not know that substance abuse is dangerous to health and a few male (9%, *n* = 13) and female (7%, *n* = 13) students were unsure.

The majority of both male (62%, *n* = 93) and female (60%, *n* = 107) learners were unaware of anyone who was willing to use substances. A few of the male (10%, *n* = 15) and female (10%, *n* = 20) learners were aware of someone who was willing to use substances, whereas the remaining learners, Male (30%, *n* = 43) and female (28%, *n* = 50) were not sure (see Table 2 for details).

**TABLE 2:** Distribution of respondents according to substance use background.

Variable	Male						Female					
	
	Yes		No		Unsure		Yes		No		Unsure	
					
	*N*	%	*N*	%	*N*	%	*N*	%	*N*	%	*N*	%
**Substance use background**												
Do you have substance users in your family?	21	13.91	108	71.52	22	14.5	32	18.08	124	70.06	21	11.86
Do your colleagues/friends use substances?	35	25.17	82	54.30	34	22.52	13	7.30	129	72.47	36	20.23
Are substances used in your school?	65	43.05	66	43.71	20	13.24	53	29.45	85	47.22	42	23.33
Are substances easy to get in your community/village?	72	48.32	45	30.20	32	21.48	69	38.54	55	30.73	55	30.73
Do you know that substance abuse is dangerous to health?	100	68.03	34	23.13	13	8.84	115	66.09	46	26.44	13	7.47
Is there someone who is willing to drugs, dagga, glue etc. at school?	15	9.93	93	61.59	43	28.48	20	11.30	107	60.45	50	28.25

## Discussion

The findings of this study showed a very low prevalence rate of substance abuse amongst the learners with only 6% male and 2% of female learners attesting to have ever used substances; this is similar to the discovery of Onya and Flisher with rural high school students in Mankweng, Limpopo Province.^12^ Also, this finding concurs with Lo et al., who observed the significantly strong effects of the protective roles of communities and schools on student substance use.^16^ Hence, the low prevalence rate in rural schools in Limpopo Province may be due to the traditional African values regarding substance abuse, especially by young people, as an indicator of a lack of proper upbringing of the child by his or her parents. The values also classify a substance user as a bad, unmannered and uncultured person. Also, most parents will discourage their children from associating with anyone using substances. Children may also not be exposed to other cultural, economic, media and modernisation factors that encourage such behaviours.^12,16^ The low prevalence rate amongst the learners may also be influenced by the knowledge that substance abuse is dangerous to the health, as is revealed in this study. However, what has been said above contradicts the 2014 USA National Institute on Drugs survey, which demonstrated a high rate of e-cigarette use and easing attitudes towards some types of drug use, particularly a decrease in perceived harm and the disapproval of marijuana use amongst teenagers.^8^

This study also revealed that there were more male substance users than females, but the gender differences were small. Similarly, a study carried out in Nigeria by Oshodi, Aina and Onajole noted non-significant statistical gender differences of substance abuse amongst secondary school learners.^13^ Likewise, Onya and Flisher, as well as Moodley and Matjila, discovered that the prevalence rate of cigarette smoking was higher amongst boys in comparison to girls.^3,12^ One probable reason given for such difference is that, in most cultures, substance abuse such as cigarette smoking is tolerated amongst males and often not tolerated amongst females, especially in black communities.^5^

In the current study, the age of debut of substance use for most of the learners falls within the adolescent period. This finding is in line with other findings across the world, including South Africa.^3^ Various studies have confirmed the adolescent age as the high risk age group for substance use.^13^ Meanwhile, some of the learners who are using substances fail to quit the habit, possibly due to the addictive nature of substances such as drugs. What is consoling is that all the female students attested to having stopped using substances.

However, the substance use background tends to have influenced or affected the learners’ behaviour positively about substance abuse, because the majority, that is 72% male and 70% female, learners said that they did not have family members who were substance users. Also, most of them do not associate with friends or colleagues who are substance users, especially the female students. Finally, and what is also worrying, is that most of the students attested to the fact that there is easy access to substances in their communities. It is, therefore, necessary for the government to reinforce legislation that addresses drug control in the country.

## Conclusion and recommendations

This study has confirmed some relevant findings from previous studies performed in Limpopo Province that the prevalence rate of substance abuse amongst rural secondary school learners is low. It has also established that most of the learners that abuse substances started during the adolescent stage. However, easy access of the learners to substances in their communities may hinder the eradication of substance abuse amongst teenagers; therefore, this needs to be addressed. Moreover, since some of the learners have failed on their own to stop using substances, there is a need to develop a counselling programme in each school to help refer such learners to rehabilitation centres, and to provide proper support for them.
